# Comprehensive catwalk gait analysis in a chronic model of multiple sclerosis subjected to treadmill exercise training

**DOI:** 10.1186/s12883-017-0941-z

**Published:** 2017-08-22

**Authors:** Danielle Bernardes, Alexandre Leite Rodrigues Oliveira

**Affiliations:** Department of Structural and Functional Biology, Institute of Biology, University of Campinas, Rua Monteiro Lobato, 255, Campinas, Sao Paulo 13.083-862 Brazil

**Keywords:** Experimental autoimmune encephalomyelitis (EAE), Walking impairment, Treadmill exercise, Catwalk (CT), Neuropathic pain

## Abstract

**Background:**

Multiple sclerosis (MS) is a demyelinating disease with a wide range of symptoms including walking impairment and neuropathic pain mainly represented by mechanical allodynia. Noteworthy, exercise preconditioning may affect both walking impairment and mechanical allodynia. Most of MS symptoms can be reproduced in the animal model named experimental autoimmune encephalomyelitis (EAE). Usually, neurological deficits of EAE are recorded using a clinical scale based on the development of disease severity that characterizes tail and limb paralysis. Following paralysis recovery, subtle motor alterations and even mechanical allodynia investigation are difficult to record, representing sequels of peak disease. The aim of the present study was to investigate the walking dysfunction by the catwalk system (CT) in exercised and non-exercised C57BL/6 mice submitted to EAE with MOG_35–55_ up to 42 days post-induction (dpi).

**Methods:**

Twenty-four C57BL/6 female mice were randomly assigned to unexercised (*n* = 12) or exercised (*n* = 12) groups. The MOG_35–55_ induced EAE model has been performed at the beginning of the fifth week of the physical exercise training protocol. In order to characterize the gait parameters, we used the CT system software version XT 10.1 (Noldus Inc., The Netherlands) from a basal time point (before induction) to 42 days post induction (dpi). Statistical analyses were performed with GraphPad Prisma 4.0 software.

**Results:**

Data show dynamic gait changes in EAE mice including differential front (FP) and hind paw (HP) contact latency. Such findings are hypothesized as related to an attempt to maintain balance and posture similar to what has been observed in patients with MS. Importantly, pre-exercised mice show differences in the mentioned gait compensation, particularly at the propulsion sub-phase of HP stand. Besides, we observed reduced intensity of the paw prints as well as reduced print area in EAE subjects, suggestive of a development of chronic mechanical allodynia in spite of being previously exercised.

**Conclusions:**

Our data suggest that Catwalk system is a useful tool to investigate subtle motor impairment and mechanical allodynia at chronic time points of the EAE model, improving the functional investigation of gait abnormalities and demyelination sequelae.

**Electronic supplementary material:**

The online version of this article (doi:10.1186/s12883-017-0941-z) contains supplementary material, which is available to authorized users.

## Background

Multiple sclerosis (MS) is the most frequent demyelinating disease worldwide, affecting around 2.3 million people. The majority of the patients (80–85%) present the remitting-recurrent clinical form with transient symptoms that are associated with inflammatory infiltrates followed by demyelination that progress chronically to permanent neurological damage [1]. As it is a disease with disseminated lesions in time and space in the central nervous system (CNS), clinical signs and symptoms of MS can be variable between patients and include mostly motor and sensory dysfunctions [2, 3].

Indeed, motor and balance disorders contribute to the progressive gait weakness, so that about 50% of patients may require ambulation support in approximately 10–20 years from diagnosis [4]. Noteworthy, the walking instability is associated with falls that lead to essential approaches aiming its prevention [5]. Also, pain affects around 63% of MS patients, and it is composed of a variety of pain syndrome, including neuropathic pain, which is present in 26% of the cases [6]. Of note, a characteristic symptom of neuropathic pain is mechanical allodynia in which an exaggerated response to non-noxious stimuli is observed, constituting a significant clinical problem [7, 8].

Some of MS symptoms as well as the histopathological hallmarks of the disease can be deeply investigated in the so-called experimental autoimmune encephalomyelitis (EAE) animal model [9]. Such studies have helped to understand the mechanisms of the disease along with the detection of some therapeutic approaches. Accordingly, the EAE model induced by myelin oligodendrocyte glycoprotein (MOG)_35–55_ peptide offers the possibility to study chronic disease, and it is a well-accepted model for testing neuroprotective strategies since it presents pronounced axonal/neuronal damage [10].

Remarkably, neurological deficits in mice subjected to EAE are classically recorded using a clinical scale with several levels of disease severity representing a broad-spectrum ranging from walking impairment to paralysis [11, 12]. However, subtle gait alterations are hard to detect after disease score stabilization in the chronic periods. Besides, neuropathic pain investigation is biased by this approach as it depends on the subjective observation of the investigator. Thus, mechanical allodynia has usually been evaluated by the von Frey filaments test in rodents [13, 14].

The catwalk automated quantitative gait analysis (CT) is a computer-assisted method for locomotor analysis in which is possible to quantify several and refined gait parameters and detect innumerous motor abnormalities [15]. In addition, different parameters investigated by CT method show a high degree of correlation with mechanical allodynia measured by von-Frey filaments test [7, 16, 17]. Quantitative gait analysis by CT method has been performed in the EAE model using Lewis rats [18] or Brown Norway rats [19]. However, von-Frey filaments method studies show hyper nociception mostly before the onset of motor dysfunction [8, 13, 14]. Also, gait analysis showed no preclinical abnormalities in EAE animals [18, 19].

Regarding strategies to prevent mechanical allodynia, exercise-preconditioning protocols aim developing neuroprotective mechanisms [20], which may have a positive impact on walking impairment [21, 22]. In this sense, a significant clinical score attenuation of EAE after specific programs of regular exercise in mice was previously reported [23, 24]. To our knowledge, specific investigation of subtle walking impairment and neuropathic pain has never been performed in exercised mice. Such approach may provide useful information regarding functional recovery and neuroprotection. Therefore, the CT system has been used in the present study aiming to investigate the walking dysfunction in exercised and non-exercised C57BL/6 mice induced to EAE with MOG_35–55_ in a chronic approach from immunization to 42 days post-induction (dpi).

## Methods

### Animals

The Multidisciplinary Center for Biological Research (CEMIB/UNICAMP, Campinas, SP, Brazil) supplied the female C57BL/6 mice (4–6 weeks old) used herein. The animals were maintained on a 12/12 h light/dark cycle and were provided with food and water ad libitum. Efforts were made to avoid any unnecessary distress to the animals, and all experiments were carried out in accordance with international guidelines and principles regulated by the National Council of Animal Experimentation (CONCEA, Brazil) for the care and use of animals. The Ethics Committee on Animal Experimentation of University of Campinas (CEUA/UNICAMP, protocol n° 3844–1) approved the protocols, and the animals were randomly assigned to unexercised (EAE; *n* = 12) or exercised (EAE-Ex; *n* = 12) groups.

### Physical exercise protocol

Based on previous work that promoted significant attenuation of EAE clinical score [24–26], we investigated whether a similar volume and duration of exercise executed in a treadmill equipment would have the same effect. For that, mice were familiarized to the motorized treadmill for five consecutive days with a progressive increase of time (5 to 25 min), a speed of 6 m/min and 11° of inclination. Next, the exercise protocol consisted of five more weeks (5 days/week) of forced running at 11 m/min, one session/day for 30 min. To account the stress associated with the environment, the unexercised mice were placed on a bench on the side of the treadmill. With this approach, the animals performed 6 weeks of pre-conditioning exercise before the onset of the EAE clinical scale.

### EAE induction and clinical assessment

The MOG_35–55_ peptide injection has been performed at the beginning of the fifth week of the physical exercise training protocol, as it has been previously standardized [25]. Accordingly, EAE was induced by subcutaneous immunization (in the tail base) with an emulsion containing 100 μg of MOG_35–55_ peptide in complete Freund’s adjuvant (CFA), supplemented with 4 mg/ml *Mycobacterium tuberculosis* H37Ra (Difco Laboratories, Detroit, MI, USA). *Bordetella pertussis* toxin (300 ng/animal; Sigma-Aldrich, St. Louis, MO, USA) was injected i.p. on the day of immunization and after 48 h. This protocol promoted the development of EAE clinical signs from 11 to 12 dpi and peaked at around 16–17 dpi, according to the clinical score assessment. To that end, animal weight and clinical score were monitored daily. The scores were defined as follows: 0 no clinical signs, 1 tail paralysis (or loss of tail tone), 2 tail paralysis and hind-limb weakness (visible paresis) and 3 one or two hind-limb paralysis.

### Gait analysis

The CatWalk system (CT) consists in an objective test to detect relevant functional changes in studies regarding regenerative strategies using the EAE model [19]. Therefore, in a first attempt to characterize the gait parameters in a chronic approach in this model, we used the CT system software version XT 10.1 (Noldus Inc., The Netherlands). The system was used to analyze gait profile in female C57BL6/J mice induced to EAE model by MOG_35–55_ peptide injection from a basal time point (before induction) to 42 days post induction (dpi). Therefore, the data were normalized against basal values, which were settled as 100% in each parameter. Of note, the study was initiated with 12 animals per group (sedentary and exercised). In the CT system method, animals must cross an illuminated walkway with a glass floor to the collection of the paw prints. However, with the disease progression, some animals were not able to cross the walkway in some of the time points. Therefore, the number of animals analyzed at each time point is described in Table 1.

**Table 1:** Animals analyzed at each time point

Time point of analysis	EAE mice	EAE-Exercised mice
Basal	12 (100%)	12 (100%)
5 dpi	12 (100%)	12 (100%)
12 dpi	12 (100%)	12 (100%)
19 dpi	07 (58%)	04 (33%)
22 dpi	10 (83%)	08 (67%)
25 dpi	08 (67%)	05 (42%)
28 dpi	10 (83%)	07 (58%)
31 dpi	06 (75%)	05 (63%)
36 dpi	07 (88%)	04 (50%)
42 dpi	07 (88%)	04 (50%)

Data are presented as absolute number (percentage)

For the analysis, each animal was placed into the walkway and was allowed to move freely in both directions with a run duration between 0.50 and 5.00 s and a maximum allowed speed variation of 60%. A high-speed camera carried out data acquisition and the software automatically classified the paw prints. The camera gain was set to 25.01 and the detection threshold to 0.25, for the detection of all parameters used in the experiments. Four compliant runs were acquired per trial and no food restriction or reward was used.

### Rotarod

**Fig. 1:**
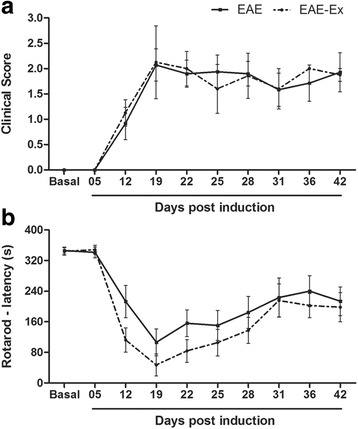
Prior treadmill exercise does not influence clinical score and rotarod results in EAE mice. No significant difference between EAE and EAE-Ex groups can be observed. **a** Clinical score. EAE (*continuous line*): Basal and 5 dpi vs 19–42 dpi (*p* < 0.0001), Basal and 5 dpi vs 12 dpi (*p* < 0.01) and 12 dpi vs 19 dpi (*p* < 0.05). EAE-Ex (*dashed line*): Basal and 5 dpi vs 12–42 dpi (*p* < 0.0001) and 12 dpi vs 19 dpi (*p* < 0.01). **b** Rotarod motor test. EAE (*continuous line*): Basal and 5 dpi vs 19 dpi (*p* < 0.0001), 22 and 25 dpi (*p* < 0.01) and 28 dpi (*p* < 0.05). EAE-Ex (*dashed line*): Basal and 5 dpi vs 12–28 dpi (*p* < 0.0001), vs 31–42 dpi (*p* < 0.05) and 19 dpi vs 31–42 dpi (*p* < 0.05)

Before collection of the data, animals were familiarized with the equipment for three consecutive days. Every session, each mouse was placed 3 times on the turning wheel for 5 min with 20–30 min of rest between the trials. The velocities (rotations per minute, rpm) were 5 rpm, 10 rpm and 16 rpm on the first, second and third day, respectively. On the fourth day, the basal time point test consisted on the acceleration from 5 to 25 rpm for 6 min (360 s), being finalized when the animal did not maintain itself on the turning wheel voluntarily, losing the balance. Two to three attempts were recorded and the average calculated for each animal. On days 5, 12, 19, 22, 25, 28, 31, 36 and 42 post induction (the same for catwalk evaluation), the animals were retested. In turn, rotarod data collections occurred always before catwalk session in the morning. As it is possible to observe in Fig. 1, almost all animals can keep walking on the turning wheel by the whole period of acceleration from 5 to 25 rpm on basal and 5 dpi time points.

### Statistical analysis

Statistical analyses were performed with GraphPad Prisma 4.0 software and data are shown as mean ± standard error. The differences between groups were considered significant when *P*-values were minor than 0.05. All data were subject to a two-way analysis of variance (ANOVA) with *Bonferroni* post-test in order to investigate the percentage of variation caused by the exercise, by the disease per se or by the interaction between these two factors. When applicable, a one-way ANOVA with *Newman-Keuls* multiple comparison tests was performed in order to investigate the effect of the disease progression, which can be observed on the figure legends (comparisons between time points). Additionally, a correlation analysis between clinical score and run duration with all the other parameters was also performed using the Pearson test.

## Results

### Regular treadmill exercise previously to EAE onset does not influence clinical score, rotarod data, and speed of the walking in EAE mice

**Fig. 2:**
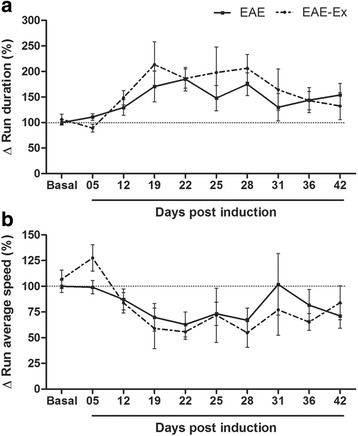
EAE animals walk slower and treadmill exercise does not improve normal gait recovery. No significant difference between EAE and EAE-Ex groups is depicted. **a** Run Duration. EAE (continuous line): Basal vs 22 dpi (*p* < 0.05). EAE-Ex (dashed line): 5 dpi vs 19–28 dpi (*p* < 0.05). **b** Run Average Speed. EAE-Ex (dashed line): 5 dpi vs 22 and 28 dpi (*p* < 0.01)

Figures 1 and 2 (and Additional file 1: raw data) demonstrate that there is no effect of the exercise approach used in the present study on the clinical score, rotarod data and speed of the walking on CT apparatus in EAE mice. For rotarod motor test data, the two-way ANOVA revealed an isolated effect of exercise (*p* < 0.05). However, there was a higher impact of the disease (*p* < 0.0001) and no interaction between exercise and EAE (*p* > 0.05) and no *Bonferroni* post hoc differences between these two groups. Similar to what has occurred to clinical score, the one-way ANOVA of rotarod data revealed some differences between time points for EAE (continuous line) and EAE-Ex (dashed line) groups (Fig 1).

Figure 2 shows the data about the gait speed and the two-way ANOVA revealed only a pronounced effect of the disease (*p* < 0.0001). On the same way, the one-way ANOVA showed some differences between time points for the two groups. Interestingly, the statistical differences were more evident in the exercised group although the correlation data presented in Table 2 are statistically similar for the two groups (EAE and EAE-Ex). Observe the values of correlation between clinical score (CS) and run duration (RD) and between rotarod (R) and CS and RD. Taken together, these data suggest that EAE animals walk slower especially around 19–28 dpi.

**Table 2:** Correlation of clinical score, run duration and rotarod with some of CT parameters

*Parameters*	*EAE*			*EAE-Ex*		
	*CS*	*RD*	*R*	*CS*	*RD*	*R*
Clinical score	−	0.73^#^	−0.73^#^	−	0.72^#^	−0.77^#^
Run Duration (s)	0.73^#^	−	−0.65^#^	0.72^#^	−	−0.64^#^
Rotarod (s)	−0.73^#^	−0.65^#^	−	−0.77^#^	−0.64^#^	−
Run Average Speed (cm/s)	−0.65^#^	−0.87^#^	0.60^#^	−0.73^#^	−0.95^#^	0.59^#^
Cadence (steps/s)	−0.67^#^	−0.88^#^	0.61^#^	−0.66^#^	−0.93^#^	0.60^#^
Regularity Index (%)	−0.65^#^	−0.67^#^	0.59^#^	−0.75^#^	−0.69^#^	0.58^#^
Phase Dispersions RF → LH	0.35^+^	0.38^+^	−0.34^+^	0.64^#^	0.51^#^	−0.34^+^
Phase Dispersions LF → RH	0.32^+^	0.29^+^	−0.22^*^	0.61^#^	0.55^#^	−0.38^+^
Couplings RF → LH	−0.69^#^	−0.67^#^	0.62^#^	−0.54^#^	−0.45^+^	0.32^+^
Couplings LF → RH	−0.63^#^	−0.50^#^	0.47^#^	−0.51^#^	−0.44^+^	0.28^*^
Diagonal Support (%)	−0.62^#^	−0.73^#^	0.48^#^	−0.62^#^	−0.75^#^	0.39^+^
Lateral Support (%)	0.51^#^	0.33^+^	−0.42^#^	0.71^#^	0.35^+^	−0.40^+^
Three Support (%)	0.36^+^	0.59^#^	−0.21^*^	0.38^+^	0.66^#^	−0.27^*^
Girdle Support (%)	0.35^+^	0.39^+^	−0.43^#^	0.30^*^	0.20	−0.22
Zero Support (%)	0.17	0.01	−0.08	0.41^+^	−0.01	−0.27^*^

Legend: ^#^ *p* < 0.0001; ^+^ *p* < 0.01; ^*^ *p* < 0.05; CS: Clinical Score; RD: Run Duration and; R: Rotarod. Data are demonstrated per group (EAE: non-exercised mice and EAE-Ex: exercised mice)

### EAE animals present decreased inter-paw coordination, and prior exercise seems to modify the style of support during the progression of the disease

**Fig. 3:**
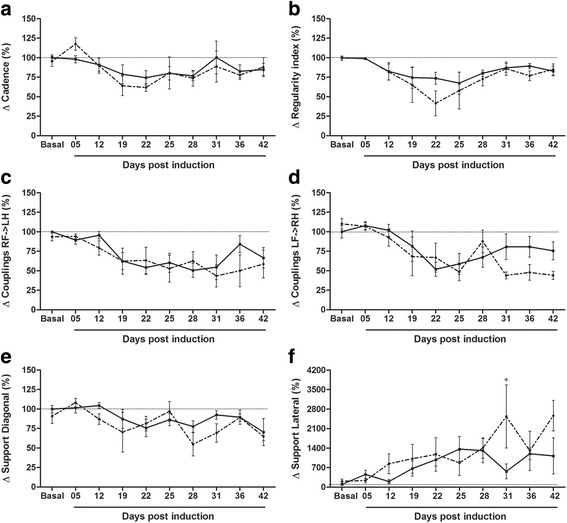
Exercised and non-exercised EAE animals present decreased inter-paw coordination. **a** Cadence. EAE-Ex (*dashed line*): 5 dpi vs 22 dpi (*p* < 0.01). **b** Regularity Index. EAE-Ex (*dashed line*): 5 dpi vs 22 dpi (*p* < 0.01). **c** Couplings RF- > LH. EAE (*continuous line*): Basal vs 22, 25 and 31 dpi (*p* < 0.05). EAE-Ex (*dashed line*): Basal and 5 dpi vs 31 dpi (*p* < 0.05). **d** Couplings LF- > RH. EAE (*continuous line*): Basal to 12 dpi vs 22 dpi (*p* < 0.05). EAE-Ex (*dashed line*): Basal and 5dpi vs 22–25 and 31–42 dpi (*p* < 0.05). **e** Support diagonal. EAE-Ex (*dashed line*): 5 dpi vs 28 dpi (*p* < 0.01). **f** Support lateral. EAE-Ex (*dashed line*): Basal and 5 dpi vs 31 and 42 dpi (*p* < 0.05). + Represents *p* < 0.05 between EAE and EAE-Ex at 31 dpi

Table 2 demonstrates, along with Fig. 3 (and Additional file 1: raw data), the inter-paw coordination such as cadence, regularity index, phase dispersions, couplings and styles of support. The two-way ANOVA revealed that there was no interaction between exercise and disease for any of these variables (*p* > 0.05). However, the correlation data in Table 2 and some distinction of the one-way ANOVA between the time points suggest a differential exercise and non-exercise modulation of the inter-paw coordination after EAE induction. An isolated effect of the disease was observed with *p* < 0.01 for cadence (steps/s) and *p* < 0.0001 for regularity index and the correlation values of these parameters with CS, RD, and R were all high and significant (Table 2). However, although the correlation values were similar for both exercised and non-exercised animals, the statistical differences between time points were more evident for the exercised group (Fig. 3).

Gait alterations may be related to phase dispersion results (Table 2), although these effects were not detected by the one-way ANOVA between time points. Besides, in spite of phase dispersion RF- > LH has been affected by the disease (*p* < 0.01, two-way ANOVA), no other effects were observed and phase dispersion LF- > RH was not affected by any of the variables herein investigated. On the other hand, couplings (both RF- > LH and LF- > RH) were strongly affected by the disease (*p* < 0.0001; two-way ANOVA) and both groups (exercise-trained and untrained) presented significant reduction of this parameter during disease progression in comparison to basal time point (Fig. 3 c, d). Interestingly, couplings LF- > RH presented significant reduction on days 31–42 in comparison to both basal and 5 dpi just for the exercised group while the non-exercised group recovered to values close to the basal line at these time points (Fig 3 d). Taken together, these data suggest that EAE animals in spite of being previously exercised present decreased inter-paw coordination.

The measure of limb support in the present study comprised diagonal, lateral, three, girdle and no support at all (zero). Of these, the disease affected support diagonal (RF-LH or LF-RH) for both exercised and non-exercised EAE mice (Table 2, *p* < 0.01, two-way ANOVA). That means EAE mice presented reduced proportion of diagonal support, which is the most used in healthy conditions (60–70% of the all types of support). This reduction was slightly more evident in the EAE-Ex (Fig 3e). The reduction of diagonal support was mainly compensated by an increase in the lateral support (RF-RH or LF-LH) that was more evident in the EAE-Ex group. Indeed, two-way ANOVA showed that there was an isolated effect of exercise on lateral support (*p* < 0.05) and, by the data presented in Table 2, we can see that exercised animals increased this type of support with the progression of the disease in a higher magnitude that the non-exercised ones. In fact, 31 dpi shows a statistical difference between the two groups (*p* < 0.05; Fig 3f). Lateral support accounts for 0–1% of the all types of support used in healthy conditions and it was increased to 5% between the non-exercised and to 9–10% between the exercised mice in the chronic phase of EAE. Support three, which represents around 20–30% of all types of support in healthy conditions, was almost unaffected by the disease although a high correlation value was observed with RD for both groups (Table 2). That means how slow the EAE animals walk so that three paws are constantly used as support. Girdle support (RF-LF or RH-LH), which represents 1–2% of all types of support used in healthy conditions, was increased to 8% around 19 dpi for the EAE group and to 7% around 28 dpi for the EAE-Ex group. The two-way ANOVA showed an isolated effect of disease (*p* < 0.01) and a moderate correlation is observed in Table 2.

### Differential modulation of dynamic and static gait parameters of front (FP) and hind paws (HP) in EAE and exercised EAE (EAE-ex) animals

**Fig. 4:**
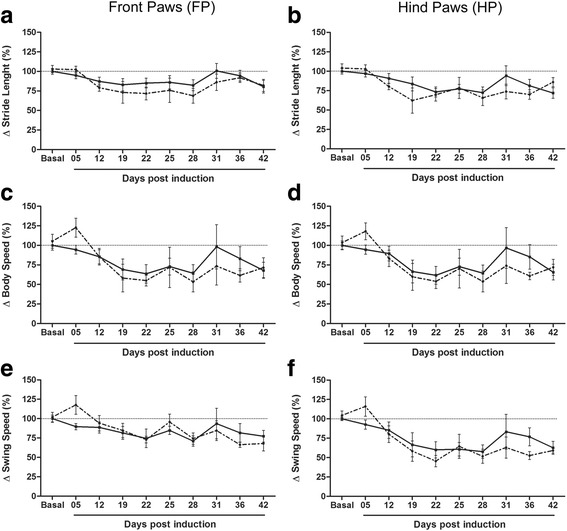
EAE animals walk slower (body and swing speed) and with short footsteps (stride length). No differences between EAE and EAE-Ex groups at any time point for any of the parameters can be observed. **a** Front paws stride length. EAE-Ex (dashed line): Basal and 5 dpi vs 22 and 28 dpi (*p* < 0.05). **b** Hind paws stride length. EAE-Ex (dashed line): Basal and 5 dpi vs 19, 22 and 28 dpi (*p* < 0.05). **c** Front paws body speed. EAE-Ex (dashed line): 5 dpi vs 22 and 28 dpi (*p* < 0.05). **d** Hind paws body speed. EAE-Ex (dashed line): 5 dpi vs 22 and 28 dpi (*p* < 0.05). **e** Front paws swing speed. No differences detected. **f** Hind paws swing speed. EAE (continuous line): Basal vs 28 dpi (*p* < 0.05). EAE-Ex (dashed line): 5 dpi vs 12–42 dpi (*p* < 0.05) and Basal vs 22 and 28 dpi (*p* < 0.05)

Overall, the remaining data are related to static and dynamic CT parameters of FP and HP of EAE and EAE-Ex mice. Unless for max contact at, the two-way ANOVA also revealed that there was no significant interaction between exercise and disease (*p* > 0.05). In fact, most of them were just related to the effect of the disease, as seen in Fig. 4 about stride length (*p* < 0.0001 for FP and HP), body speed (*p* < 0.0001 for FP and HP) and swing speed (*p* < 0.01 for FP and *p* < 0.0001 for HP). Although the one-way ANOVA has shown some effect between time points, mostly for the exercised animals, Table 3 demonstrates similar correlation values between these parameters and CS and RD for both groups. Noteworthy, as these results are strongly affected by run duration, the statistical differences between time points were more evident for the EAE-Ex group.

**Table 3:** Correlation of clinical score and run duration with other CT parameters

Parameters	Front paws (FP)				Hind paws (HP)			
	EAE		EAE-Ex		EAE		EAE-Ex	
	CS	RD	CS	RD	CS	RD	CS	RD
Stride Length (cm)	−0.60^#^	−0.84^#^	−0.74^#^	−0.80^#^	−0.72^#^	−0.85^#^	−0.68^#^	−0.78^#^
Body Speed (cm/s)	−0.67^#^	−0.89^#^	−0.74^#^	−0.95^#^	−0.69^#^	−0.89^#^	−0.75^#^	−0.95^#^
Swing Speed (cm/s)	−0.51^#^	−0.71^#^	−0.42^+^	−0.63^#^	−0.73^#^	−0.85^#^	−0.75^#^	−0.83^#^
Step Cycle (s)	0.64^#^	0.82^#^	0.45^#^	0.77^#^	0.58^#^	0.88^#^	0.44^#^	0.73^#^
Stand (s)	0.70^#^	0.93^#^	0.57^#^	0.88^#^	0.18	0.43^#^	0.06	0.45^#^
Duty Cycle (%)	0.56^#^	0.81^#^	0.64^#^	0.85^#^	−0.30^+^	−0.14	−0.36^+^	0.02
Initial Dual Stance (s)	0.66^#^	0.90^#^	0.63^#^	0.88^#^	0.07	0.37^+^	0.06	0.53^#^
Terminal Dual Stance (s)	0.67^#^	0.90^#^	0.64^#^	0.87^#^	0.16	0.47^#^	0.11	0.57^#^
Base of Support (cm)	0.58^#^	0.61^#^	0.72^#^	0.73^#^	−0.31^+^	−0.18	−0.44^+^	−0.20
Max Contact Max Intensity	−0.13	0.15	−0.46^#^	−0.15	−0.55^#^	−0.36^+^	−0.76^#^	−0.47^#^
15 Most Intense Pixels	−0.08	0.20	−0.31^+^	−0.02	−0.53^#^	−0.28^+^	−0.73^#^	−0.46^#^
Max Intensity At (%)	0.51^#^	0.40^#^	0.52^#^	0.43^#^	−0.64^#^	−0.37^+^	−0.73^#^	−0.54^#^
Max Contact At (%)	0.25^*^	0.38^#^	0.44^#^	0.58^#^	−0.44^#^	−0.39^+^	−0.25^*^	−0.27^*^
Print Width (cm)	−0.40^#^	−0.28^+^	−0.66^#^	−0.42^+^	−0.51^#^	−0.31^+^	−0.41^+^	−0.20
Print Length (cm)	−0.16	−0.02	−0.29^*^	−0.07	−0.18	−0.03	−0.26^*^	−0.09
Print Area (cm^2^)	−0.15	0.11	−0.36^*^	−0.10	−0.27^*^	−0.05	−0.45^#^	−0.22

Legend: ^#^ *p* < 0.0001; ^+^ *p* < 0.01; ^*^ *p* < 0.05; CS: Clinical Score; RD: Run Duration. Data are demonstrated per group and per pair of paws

**Fig. 5:**
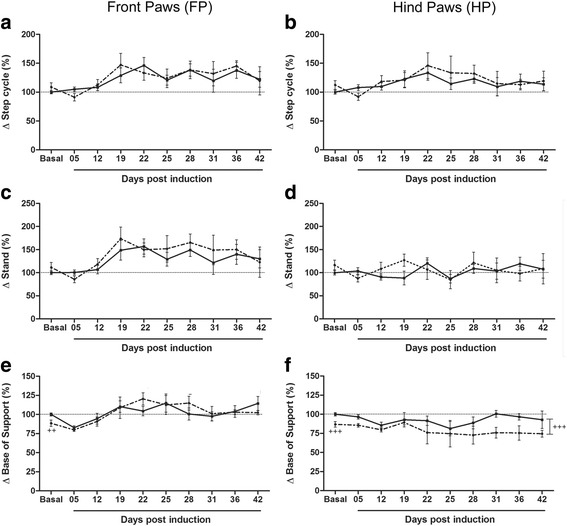
EAE animals present increased stance phase of step cycle for front paws. **a** Front paws step cycle. EAE (continuous line): Basal and 5 dpi vs 22 dpi (*p* < 0.05). **b** Hind paws step cycle. No differences detected. **c** Front paws stand. EAE-Ex (dashed line): 5 dpi vs 19 and 28 dpi (*p* < 0.05). **d** Hind paws stand. No differences detected. **e** Front paws base of support. EAE (continuous line): 5 dpi vs 25 and 42 dpi (*p* < 0.050). EAE-Ex (dashed line): 5 dpi vs 22–28 dpi (*p* < 0.05) and Basal and 12 dpi vs 22 dpi (*p* < 0.05). ++ Represents *p* < 0.01 between EAE and EAE-Ex at Basal time point. **f** Hind paws base of support. ^+++^ *p* < 0.0001 between the curves (EAE vs EAE-Ex) by student T test

Table 3 (and Additional file 1: raw data) shows that the disease and the reduced gait speed have increased parameters related to the stance of the FP such as step cycle, stand, duty cycle, initial and terminal dual stance. It is note worth that stand/stance is the duration in seconds of contact while swing is the duration in seconds of no contact of a paw with the glass plate. Therefore, step cycle represents the sum of swing and stand duration while duty cycle represents stand as a percentage of step cycle. Dual stand/stance is the duration in seconds of contact for both limbs simultaneously (hind or forelimbs). Therefore, initial and terminal dual stance are, respectively, the first and the second time in a step cycle of a paw and its contralateral when making contact with the glass plate. All of these parameters for FP were also strongly affected by the disease (two-way ANOVA, *p* < 0.0001; Fig. 5 a and c). These data suggest that EAE animals keep FP more time on the glass while walking. On the other hand, only step cycle for hind paws were positive and significantly correlated with CS and RD for both groups and the two-way ANOVA shows an isolated effect of disease (*p* < 0.05). The disease also affected duty cycle for hind paws (*p* < 0.01), but the correlation data shows a moderate and negative relationship between them (Table 3). Noteworthy, stand, initial and terminal dual stance for hind paws were not affected by the disease, although some positive correlation with RD can be observed in Table 3. Taken together, this differential FP and HP response may suggest a compensation mechanism in order to maintain gait and balance with a shift of the center of gravity to FP because of the overt dysfunction of HP.

A differential response from the EAE condition between FP and HP was also observed for the base of support (BOS) data (Table 3; Fig. 5 e-f). In this case, highly positive correlations with CS and RD are observed for FP with a strong effect from disease (*p* < 0.0001; two-way ANOVA), starting after 22 dpi (Fig. 5 e). Since this parameter is related to the perpendicular distance of both paws to each other, these analyses demonstrate that EAE animals walk with a wider distance between the front paws. Curiously, unpaired t-test analysis for basal time point (before induction) demonstrates that 6 weeks of treadmill exercise decreased the distance between the left and the right FP during the walking (*p* < 0.05; sedentary: 1.42 ± 0.03 cm and exercised: 1.26 ± 0.06 cm). However, this basal difference did not influence the development of wider BOS of FP after EAE induction.

**Fig. 6:**
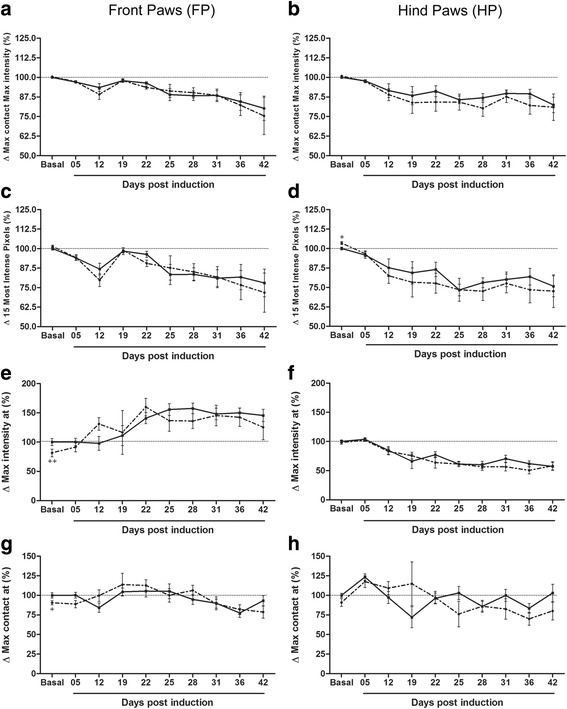
EAE animals present reduced intensity of paw prints at chronic disease. ^+^ *p* < 0.05 and ^++^ *p* < 0.01 between EAE and EAE-Ex groups. **a** Front paws max contact max intensity. EAE (continuous line): Basal vs 36 dpi (*p* < 0.05) and Basal to 22 dpi vs 42 dpi (*p* < 0.05). EAE-Ex (dashed line): Basal vs 36 dpi (*p* < 0.05) and Basal, 5, 19 and 22 dpi vs 42 dpi (*p* < 0.05). **b** Hind paws max contact max intensity. EAE (continuous line): Basal vs 25, 28 and 42 dpi (*p* < 0.05) and 5 dpi vs 42 dpi. EAE-Ex (dashed line): Basal vs 28 and 42 dpi (*p* < 0.05) and 5 dpi vs 28 dpi. **c** Front paws mean intensity of the 15 most intense pixels. EAE (continuous line): Basal vs 42dpi (*p* < 0.05). EAE-Ex (dashed line): Basal vs 12, 36 and 42 dpi (*p* < 0.05). **d** Hind paws mean intensity of the 15 most intense pixels. EAE (continuous line): Basal and 5 dpi vs 25, 28 and 42 dpi (*p* < 0.05). EAE-Ex (dashed line): Basal vs 12–42 dpi (*p* < 0.05) and 5 dpi vs 28 dpi (*p* < 0.05). + Represents *p* < 0.05 between EAE and EAE-Ex at Basal time point. **e** Front paws max intensity at. EAE (continuous line): Basal, 5 and 12 dpi vs 22–42 dpi (*p* < 0.05) and 19 dpi vs 28 dpi (*p* < 0.05). EAE-Ex (dashed line): Basal vs 22 and 31 dpi (*p* < 0.05) and 5 dpi vs 31 dpi (*p* < 0.01). ++ Represents *p* < 0.01 between EAE and EAE-Ex at Basal time point. **f** Hind paws max intensity at. EAE (continuous line): Basal and 5 dpi vs 19–42 dpi (*p* < 0.01) and 12 dpi vs Basal, 5, 25, 28, 36 and 42 dpi (*p* < 0.05). EAE-Ex (dashed line): Basal and 5 dpi vs 12–42 dpi (*p* < 0.05) and 12 dpi vs 22–42 dpi (*p* < 0.05). **g** Front paws max contact at. + Represents *p* < 0.05 between EAE and EAE-Ex at Basal time point. **h** Hind paws max contact at. EAE (continuous line): 5 dpi vs 19, 28 and 36 dpi

**Fig. 7:**
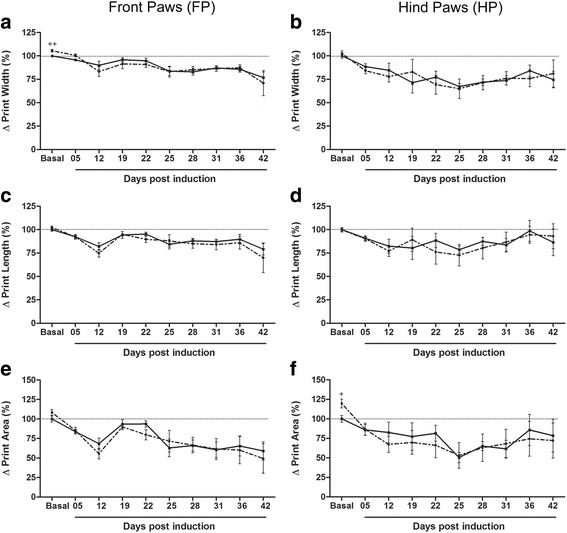
EAE animals present decreased contact area of the paw prints at the chronic disease. **a** Front paws print width. EAE (continuous line): Basal vs 25, 28, 36 and 42 dpi (*p* < 0.05), 19 dpi vs 12 and 42 dpi (*p* < 0.05) and 5 and 22 dpi vs 42 dpi. EAE-Ex (dashed line): Basal vs 12, 25–31 and 42 dpi (*p* < 0.05) and 5 dpi vs 12 and 42 dpi. ++ Represents *p* < 0.01 between EAE and EAE-Ex at Basal time point. **b** Hind paws print width. EAE (continuous line): Basal vs 19, 25 and 28 dpi (*p* < 0.05). EAE-Ex (dashed line): Basal vs 22–28 dpi (*p* < 0.05). **c** Front paws print length. EAE (continuous line): Basal vs 12, 25 and 42 dpi (*p* < 0.05). EAE-Ex (dashed line): Basal vs 12 and 42 dpi (*p* < 0.01). **d** Hind paws print length. No differences detected. **e** Front paws print area. EAE (continuous line): Basal vs 12 and 25–42 dpi (*p* < 0.05). EAE-Ex (dashed line): Basal vs 05–12 and 22–42 dpi. **f** Hind paws print area. EAE-Ex (dashed line): Basal vs 25–28 dpi (*p* < 0.05). + Represents *p* < 0.05 between EAE and EAE-Ex at Basal time point

Detailed unpaired t-test analysis for basal time point of all parameters revealed several differences, suggesting some effect of exercise on the walking pattern. For example, exercise increased FP print width (Fig. 7 a; *p* < 0.01; sedentary: 0.74 ± 0.006 cm and exercised: 0.78 ± 0.010 cm) but reduced its max intensity at (Fig. 6 e; *p* < 0.01; sedentary: 31.68 ± 1.91% and exercised: 24.19 ± 1.42%) and its max contact at (Fig. 6 g; *p* < 0.05; sedentary: 37.27 ± 1.26% and exercised: 33.73 ± 0.99%). In its way, exercise increased HP print area (Fig. 7 F; *p* < 0.05; sedentary: 0.32 ± 0.014 cm and exercised: 0.39 ± 0.018 cm) and its mean intensity of the 15 most intense pixels (Fig. 6 d; *p* < 0.05; sedentary: 221.7 ± 1.98 and exercised: 229.4 ± 2.09 cm). Analogous to BOS of FP, these last basal alterations did not influence the course of each parameter during disease.

Similarly to BOS of FP, hind paws were positioned with a narrower distance after 6 weeks of exercise (basal time point on Fig. 5 f; *p* < 0.0001; sedentary: 2.5 ± 0.06 cm and exercised: 2.2 ± 0.09 cm), meaning that 6 weeks of treadmill exercise decrease the BOS for FP and HP of mice in healthy condition. However, for BOS of the HP, a detached curve of exercised from sedentary mice is visible on the graph and the effect of exercise is quite significant during all the period studied (Fig. 5 f; *p* < 0.0001; two-way ANOVA). No effects from disease were observed either by the two- or one-way ANOVA and a negative correlation were observed (Table 3), suggesting that EAE animals may decrease the distance between the hind paws over the walking dysfunction. Therefore, the overall response of BOS between FP and HP were opposite during disease progression, which, along with data about stance, is suggestive of a compensation mechanism.

M*ax intensity at* (Fig. 6 e-f) and *max contact at* (Fig. 6 g-h) were also altered in an opposite manner with overall positive correlations with CS and RD for FP while negative values were observed for the HP (Table 3). This discrepancy is quite visible on the graphs of *max intensity at*, which was affected by the disease with (*p* < 0.0001) for both FP and HP and had several overt differences between the time points with a noticeable increase for FP after 22 dpi and decrease for HP after 12 dpi. Similar results were observed with max contact at even though two important dissimilarities are worth mentioning. Firstly, two-way ANOVA revealed an effect of the disease (*p* < 0.01) for both FP and HP and a significant interaction between exercise and disease for HP (*p* < 0.05). Secondly, this interaction effect caused reduced correlation values with CS and RD and no difference between the time points for the EAE-Ex group. Therefore, it seems that 6 weeks of treadmill exercise has attenuated the variations on max contact at of HP caused by the EAE induction. In other words, our data suggest that EAE mice show some fluctuations on the time spent for the transition of braking into propulsion sub-phase of stand during disease progression and it seems that prior treadmill exercise attenuates that changings for HP.

The present correlation data suggest that exercise may have had a negative synergistic effect with the disease for max contact max intensity and mean intensity of the 15 most intense pixels (Table 3). For FP only the exercised mice showed a significant and negative correlation between clinical score and gait parameters, although the two-way ANOVA revealed a strong effect of the disease (*p* < 0.0001) In turn one-way ANOVA indicated several significant differences between time points for both groups. For HP, both groups demonstrated a significant and negative correlation between these parameters and both CS and RD. However, the disease demonstrated the highest effect (*p* < 0.0001) and, one-way ANOVA presented several significant differences between time points for both groups. Taken together, these data suggest that EAE animals present reduced intensity of paw prints from 12 dpi to 42 dpi and that treadmill exercise may have strengthened this result.

In the same way, print width, print length, and print area were decreased with the progression of the disease (Fig. 7). Exercise strengthened such results, since the highest values of negative correlation between these parameters and CS were observed for the EAE-Ex group (Table 3). However, there was no interaction or isolated effect of exercise and FP was strongly affected by the disease from 12 dpi (*p* < 0.0001, one-way ANOVA). The hind paws were also affected but with variable values of P (print width: *p* < 0.0001; print length: *p* < 0.05; print area: *p* < 0.01). These data suggest that EAE animals present decreased contact area of the paw prints at chronic disease, especially for FP.

## Discussion

Several studies using treadmill exercise performed during short periods of training (2–25 days) have shown none or undersized effects on development and progression of clinical signs in EAE animals [27–31]. Therefore, we chose to investigate volume and duration of exercise similar to the protocol of swimming we have used previously in which we observed an important clinical score attenuation [24, 25].

We used regular treadmill exercise in this study in order to prepare animals in a similar ability to what they would be evaluated such as the CT system and motor rotarod, in which animals are forced to walk on the apparatus. Indeed, the basal data of the present work show several subtle motor alterations that represent an isolated effect of exercise on gait pattern in healthy condition, before the EAE induction. First, 6 weeks of treadmill exercise significantly decreased the BOS for FP and HP irrespective to the body weight. Based upon some data with animal models of ataxic gait such as cerebral ischemia [32], thyroid system dysfunction [33] and Leigh disease [34], in which an increased BOS was observed, we may suggest that exercised mice presented an increased gait stability before EAE induction.

In addition, exercised mice showed increased FP print width (Fig 7 a) with reduced both max intensity at and max contact at, suggesting that exercised mice anticipate the shifting of brake to propulsion sub-phase of stand for FP. On the other hand, an increased HP print area with increased mean intensity of the 15 most intense pixels at basal time point was observed for the exercised group, suggesting a compensation of the pressure between FP and HP. These subtle and prior adaptations caused by the exercise may have affected some of the gait parameters during the progression of the disease. First, it seems that 6 weeks of treadmill exercise have attenuated the fluctuations on the time spent for the transition of braking into propulsion sub-phase of the stand of HP. Second, BOS of HP was decreased during the studied period for the EAE-Ex group in comparison to the EAE group. These data are in agreement with evidence showing that repeated treadmill-walking tests increase gait stability in mice that is maybe associated to a habituation response to the dynamic daily feedbacks from proprioceptive, vestibular, and visual inputs [35]. However, unless for some negative effects related to neuropathic pain that will be discussed later, these subtle effects of the protocol of exercise used herein had no significant impact on EAE walking dysfunction.

Literature has shown greater effects of swimming over treadmill running on elevation of serum adrenocorticotropic hormone and corticosterone [36]; activation of sympathetic nervous system [37] increase of cell proliferation in the hippocampal dentate gyrus [38] and even preservation of spinal motoneurons [39]. Perhaps, swimming promotes specific neuroimmune modulation that counts in favor of a clinical score attenuation of EAE mice that may not be depicted with the forced running approach.

Noteworthy, our results show that EAE animals present decreased inter-paw coordination, a wider distance of perpendicular support between FP and a reduced intensity of the paw prints as well as reduced print area when compared to the basal time point. These alterations were evident until 42 dpi. In a transient way, especially from 19 to 28 dpi, EAE animals walk slower, with shorter footsteps (decreased stride length) and an increased time of FP stand, suggesting the development of a compensation mechanism to maintain gait and balance during periods of more significant walking dysfunction. Reduced velocity of movement has been demonstrated in EAE mice at 4 dpi and it was associated with the stress caused by the injections necessary to the induction procedure [40]. The speed of the walking is an important parameter of debate in walking dysfunctions since it can affect other dynamic gait parameters [16, 41–43]. For example, it is classically recognized that decreased speed of walking leads to increased period of paw contact [44] and that hind paws are much less variable with velocity than the front paws [45].

Accordingly, EAE animals keep FP more time on the glass because of the delayed time to shift brake into propulsion sub-phase of stand and that was accompanied by increased BOS, i.e., wide perpendicular distance between FP. These results were observed especially after 22 dpi with no positive effect of exercise pre-training. Overall, the present evidences that EAE mice try to keep posture and balance, by changing the center of gravity to the FP, as illustrated by positive phase dispersion and the more negative values of couplings during the progression of the disease [43].

Of note, reduction of the regularity index has been observed in the rat model of EAE [18]. In addition, rotarod motor test data presented correlation values with gait parameter similar to the ones observed with the clinical score, illustrating the important walking dysfunction during the course of the disease [46]. EAE animal’s shorter footsteps provide additional confirmation of the altered gait pattern observed herein. In this sense, a similar result has been established on 15 and 20 dpi in EAE mice [47]. Besides, reduced stride length was also observed in a mouse model of pyramidotomy [48] and in a model of reduced levels of striatal dopaminergic neurons [49].

In fact, damage to both corticospinal tract [50] and striatum [51], as well as alteration of the dopaminergic system [52], has been observed in EAE animals. In addition, reduced levels of dopamine have been associated with lower velocities of walking [53] as well as dysfunction of locomotor circuits in the lumbar spinal cord [54]. Recently, Fiander and colleagues demonstrated that EAE mice present changed angle and range of motion for hip, knee and ankle joints suggesting that these deficits are caused by reduced corticospinal axon conduction as well as reduced synchronization of the activities from the basal ganglia [12]. In turn, our study suggests that reduced stride length may be also related to a damaged corticospinal tract or even reduced dopaminergic activity caused by the disease.

It is important to highlight that EAE-Ex group presented some worsened gait parameters such as those related to intensity and contact area of the paw prints, especially FP. Such reduction started at 12 dpi and resulted in a decreased print area, what may be associated to mechanical allodynia [7, 15, 17]. Pain enhancement can be associated with demyelinating lesions, axonal damage, and release of pro-inflammatory cytokines, activated glial cells as well as excitatory neurotransmission that are characteristic of EAE development [8]. Exercising may lead to pro-inflammatory responses, especially if performed close to the onset of the disease.

## Conclusions

The great magnitude of alterations on gait parameters observed herein, during the course of EAE may be related to the fact that several areas of the CNS are affected in this model as is observed in patients with multiple sclerosis (MS). The present study demonstrates for the first time that walking speed, stride length, inter-paw coordination, intensity and area of the paw prints, base of support as well as stand are relevant parameters for gait monitoring during EAE. Such parameters can in turn be used to evaluate new treatments that result in motor preservation/recovery.

## Additional file

Additional file 1: Raw Data. Complete list of all original data generated during this study. (XLSX 117 kb)

## Abbreviations

ANOVA: Analysis of variance
BOS: Base of support
CEMIB/UNICAMP: Multidisciplinary Center for Biological Research of University of Campinas
CEUA/UNICAMP: Ethics Committee on Animal Experimentation of University of Campinas
CFA: Complete Freund’s adjuvant
CNS: Central nervous system
CONCEA: National Council of Animal Experimentation
CS: Clinical score
CT: Catwalk
Dpi: Days post induction
EAE: Experimental autoimmune encephalomyelitis
EAE-Ex: EAE animals submitted to prior regular exercise
FP: Front paws
HP: Hind paws
LF- > RH: Left front to right hind
MOG: Myelin oligodendrocyte glycoprotein
MS: Multiple sclerosis
R: Rotarod
RD: Run duration
RF- > LH: Right front to left hind
rpm: Rotations per minute

## References

[1] Loleit V, Biberacher V, Hemmer B (2014). Current and future therapies targeting the immune system in multiple sclerosis. Curr Pharm Biotechnol.

[2] Bagnato F, Centonze D, Galgani S, Grasso MG, Haggiag S, Strano S (2011). Painful and involuntary multiple sclerosis. Expert Opin Pharmacother.

[3] Doshi A, Chataway J (2016). Multiple sclerosis, a treatable disease. Clin Med (Northfield Il).

[4] Motl RW, Goldman MD, Benedict RHB (2010). Walking impairment in patients with multiple sclerosis: exercise training as a treatment option. Neuropsychiatr Dis Treat.

[5] Comber L, Galvin R, Coote S (2017). Gait deficits in people with multiple sclerosis: a systematic review and meta-analysis. Gait Posture.

[6] Foley PL, Vesterinen HM, Laird BJ, Sena ES, Colvin LA, Chandran S (2013). Prevalence and natural history of pain in adults with multiple sclerosis: systematic review and meta-analysis. Pain.

[7] Vrinten DH, Hamers FFT (2003). “CatWalk” automated quantitative gait analysis as a novel method to assess mechanical allodynia in the rat; a comparison with von Frey testing. Pain.

[8] Khan N, Smith MT (2014). Multiple sclerosis-induced neuropathic pain: pharmacological management and pathophysiological insights from rodent EAE models. Inflammopharmacology.

[9] Lühder F, Gold R, Flügel A, Linker RA. Brain-derived neurotrophic factor in neuroimmunology: lessons learned from multiple sclerosis patients and experimental autoimmune encephalomyelitis models. Arch Immunol Ther Exp. 2013; 61:95–105. 10.1007/s00005-012-0211-0 23283517

[10] Lassmann H, Bradl M (2017). Multiple sclerosis: experimental models and reality. Acta Neuropathol.

[11] Recks MS, Addicks K, Kuerten S (2011). Spinal cord histopathology of MOG peptide 35-55-induced experimental autoimmune encephalomyelitis is time- and score-dependent. Neurosci Lett.

[12] Fiander MDJ, Stifani N, Nichols M, Akay T, Robertson GS (2017). Kinematic gait parameters are highly sensitive measures of motor deficits and spinal cord injury in mice subjected to experimental autoimmune encephalomyelitis. Behav Brain Res.

[13] Rodrigues DH, Sachs D, Teixeira AL (2009). Mechanical hypernociception in experimental autoimmune encephalomyelitis. Arq Neuropsiquiatr.

[14] Rodrigues DH, Leles BP, Costa VV, Miranda AS, Cisalpino D, Gomes DA (2016). IL-1b is involved with the generation of pain in experimental autoimmune encephalomyelitis. Mol Neurobiol.

[15] Chen Y-J, Cheng F-C, Sheu M-L, Su H-L, Chen C-J, Sheehan J (2014). Detection of subtle neurological alterations by the catwalk XT gait analysis system. J Neuroeng Rehabil.

[16] Gabriel AF, Marcus MAE, Honig WMM, Walenkamp GHIM, Joosten EAJ (2007). The CatWalk method: a detailed analysis of behavioral changes after acute inflammatory pain in the rat. J Neurosci Methods.

[17] Pitzer C, Kuner R, Tappe-theodor A (2016). Voluntary and evoked behavioral correlates in inflammatory pain conditions under different social housing conditions. Mol Pain.

[18] Silva GAA, Pradella F, Moraes A, Farias A, dos Santos LMB, de Oliveira ALR (2014). Impact of pregabalin treatment on synaptic plasticity and glial reactivity during the course of experimental autoimmune encephalomyelitis. Brain Behav.

[19] Herold S, Kumar P, Jung K, Graf I, Menkhoff H, Schulz X, et al. CatWalk gait analysis in a rat model of multiple sclerosis. BMC Neurosci. BioMed Central; 2016; 17:78. 10.1186/s12868-016-0317-0 PMC5131412 27903258

[20] Basso DM, Hansen CN (2011). Biological basis of exercise-based treatments: spinal cord injury. Phys Med Rehabil.

[21] Pearson M, Dieberg G, Smart N (2015). Exercise as a therapy for improvement of walking ability in adults with multiple sclerosis: a meta-analysis. Arch Phys Med Rehabil.

[22] Jung SY, Kim DY, Yune TY, Shin DH, Baek S Bin, Kim CJ. Treadmill exercise reduces spinal cord injury-induced apoptosis by activating the PI3K/Akt pathway in rats. Exp Ther Med. 2014; 7:587–93. 10.3892/etm.2013.1451 PMC3919853 24520250

[23] Rossi S, Furlan R, De Chiara V, Musella A, Lo Giudice T, Mataluni G (2009). Exercise attenuates the clinical, synaptic and dendritic abnormalities of experimental autoimmune encephalomyelitis. Neurobiol Dis.

[24] Bernardes D, Brambilla R, Bracchi-Ricard V, Karmally S, Dellarole A, Carvalho-Tavares J (2016). Prior regular exercise improves clinical outcome and reduces demyelination and axonal injury in experimental autoimmune encephalomyelitis. J Neurochem.

[25] Bernardes D, Oliveira-Lima OC, da Silva TV, Faraco CCF, Leite HR, Juliano MA (2013). Differential brain and spinal cord cytokine and BDNF levels in experimental autoimmune encephalomyelitis are modulated by prior and regular exercise. J Neuroimmunol.

[26] Bernardes D, Oliveira-Lima OC, da Silva TV, Juliano MA, Dos Santos DM, Carvalho-Tavares J (2016). Metabolic alterations in experimental autoimmune encephalomyelitis in mice: effects of prior physical exercise. Neurophysiology.

[27] Le Page C, Ferry A, Rieu M (1994). Effect of muscular exercise on chronic relapsing experimental autoimmune encephalomyelitis. J Appl Physiol.

[28] Le Page C, Bourdoulous S, Béraud E, Couraud PO, Rieu M, Ferry A (1996). Effect of physical exercise on adoptive experimental auto-immune encephalomyelitis in rats. Eur J Appl Occup Physiol.

[29] Patel DI, White LJ (2013). Effect of 10-day forced treadmill training on neurotrophic factors in experimental autoimmune encephalomyelitis. Appl Physiol Nutr Metab.

[30] Wens I, Dalgas U, Verboven K, Kosten L, Stevens A, Hens N (2015). Impact of high intensity exercise on muscle morphology in EAE rats. Physiol Res.

[31] Patel DI, White LJ, Lira VA, Criswell DS, Physiology A (2016). Forced exercise increases muscle mass in EAE despite early onset of disability. Physiol Res.

[32] Parkkinen S, Ortega FJ, Kuptsova K, Huttunen J, Tarkka I, Jolkkonen J (2013). Gait impairment in a rat model of focal cerebral ischemia. Stroke Res Treat.

[33] Bárez-López S, Bosch-García D, Gómez-Andrés D, Pulido-Valdeolivas I, Montero-Pedrazuela A, Obregon MJ (2014). Abnormal motor phenotype at adult stages in mice lacking type 2 deiodinase. PLoS One.

[34] de Haas R, Russel FG, Smeitink JA (2016). Gait analysis in a mouse model resembling Leigh disease. Behav Brain Res.

[35] Wooley CM, Xing S, Burgess RW, Cox GA, Seburn KL (2009). Age, experience and genetic background influence treadmill walking in mice. Physiol Behav.

[36] Contarteze RVL, Manchado FDB, Gobatto CA, De Mello MAR (2008). Stress biomarkers in rats submitted to swimming and treadmill running exercises. Comp Biochem Physiol - A Mol Integr Physiol.

[37] Baptista S, Piloto N, Reis F, Teixeira-de-Lemos E, Garrido AP, Dias A (2008). Treadmill running and swimming imposes distinct cardiovascular physiological adaptations in the rat: focus on serotonergic and sympathetic nervous systems modulation. Acta Physiol Hung.

[38] Ra S-M, Kim H, Jang M-H, Shin M-C, Shin M-C, Lee T-H (2002). Treadmill running and swimming increase cell proliferation in the hippocampal dentate gyrus of rats. Neurosci Lett.

[39] Deforges S, Branchu J, Biondi O, Grondard C, Pariset C, Lécolle S (2009). Motoneuron survival is promoted by specific exercise in a mouse model of amyotrophic lateral sclerosis. J Physiol.

[40] Sheridan GK, Dev KK (2014). Targeting S1P receptors in experimental autoimmune encephalomyelitis in mice improves early deficits in locomotor activity and increases ultrasonic vocalisations. Sci Rep.

[41] Deumens R, Jaken RJP, Marcus MAE, Joosten EAJ (2007). The CatWalk gait analysis in assessment of both dynamic and static gait changes after adult rat sciatic nerve resection. J Neurosci Methods.

[42] Herbin M, Hackert R, Gasc JP, Renous S (2007). Gait parameters of treadmill versus overground locomotion in mouse. Behav Brain Res.

[43] Batka RJ, Brown TJ, Mcmillan KP, Meadows RM, Jones KJ, Haulcomb MM (2014). The need for speed in rodent locomotion analyses Richard. Anat Rec.

[44] Górska T, Majczyński H, Zmysłowski W (1998). Overground locomotion in intact rats: contact electrode recording. Acta Neurobiol Exp (Wars).

[45] Clarke K, Still J (1999). Gait analysis in the mouse. Physiol Behav.

[46] Moore S, Khalaj AJ, Patel R, Yoon J, Ichwan D, Hayardeny L (2014). Restoration of axon conduction and motor deficits by therapeutic treatment with glatiramer acetate. J Neurosci Res.

[47] Mitra NK, Bindal U, Hwa WE, Chua CLL, Tan CY (2015). Evaluation of locomotor function and microscopic structure of the spinal cord in a mouse model of experimental autoimmune encephalomyelitis following treatment with syngeneic mesenchymal stem cells. Int J Clin Exp Pathol.

[48] Starkey ML, Barritt AW, Yip PK, Davies M, Hamers FPT, McMahon SB (2005). Assessing behavioural function following a pyramidotomy lesion of the corticospinal tract in adult mice. Exp Neurol.

[49] Guillot TS, Asress SA, Richardson JR, Glass JD, Miller GW (2008). Treadmill gait analysis does not detect motor deficits in animal models of Parkinson’s disease or amyotrophic lateral sclerosis. J Mot Behav.

[50] Liu Z, Li Y, Zhang J, Elias S, Chopp M (2008). Evaluation of corticospinal axon loss by fluorescent dye tracing in mice with experimental autoimmune encephalomyelitis. J Neurosci Methods.

[51] Centonze D, Muzio L, Rossi S, Cavasinni F, De Chiara V, Bergami A (2009). Inflammation triggers synaptic alteration and degeneration in experimental autoimmune encephalomyelitis. J Neurosci.

[52] Gentile A, Fresegna D, Federici M, Musella A, Rizzo FR, Sepman H (2015). Dopaminergic dysfunction is associated with IL-1b-dependent mood alterations in experimental autoimmune encephalomyelitis. Neurobiol Dis.

[53] Serradj N, Jamon M (2009). The adaptation of limb kinematics to increasing walking speeds in freely moving mice 129/Sv and C57BL/6. Behav Brain Res.

[54] Koblinger K, Füzesi T, Ejdrygiewicz J, Krajacic A, Bains JS, Whelan PJ (2014). Characterization of A11 neurons projecting to the spinal cord of mice. PLoS One.

